# A ketogenic diet improves memory in females in the APOE4 mouse model of Alzheimer’s disease

**DOI:** 10.1007/s11357-025-01998-8

**Published:** 2025-11-24

**Authors:** Jacopo Di Lucente, Jennifer M. Rutkowsky, Alfredo Errico Provenzano, Giuseppe Persico, Zeyu Zhou, Lee-Way Jin, Jon J. Ramsey, Claire B. Montgomery, Kyoungmi Kim, Marco Giorgio, Izumi Maezawa, Gino A. Cortopassi

**Affiliations:** 1 Department of Pathology and MIND Institute, University of California Davis Medical Center, Sacramento, CA 95817 USA; 2 Department of Molecular Biosciences, School of Veterinary Medicine, University of California Davis, Davis, CA 95616 USA; 3 Department of Experimental Oncology, IEO, European Institute of Oncology IRCCS, 20139 Milan, Italy; 4 Department of Biomedical Sciences, University of Padova, 35131 Padua, Italy; 5 Alzheimer’s Disease Research Center, University of California Davis Medical Center, Sacramento, CA 95817 USA; 6 Department of Public Health Sciences, School of Medicine, University of California Davis, Davis, CA 95616 USA

**Keywords:** Alzheimer’s disease, APOE, Ketogenic diet, Memory, Inflammation, Cognition

## Abstract

The ε4 allele of apolipoprotein E (APOE4) is the strongest genetic risk factor for Alzheimer’s disease (AD), increasing AD risk about fourfold in ~ 34 million American and ~ 75 million European females. APOE4 carriers exhibit cerebral metabolic deficits decades before clinical onset. We previously demonstrated that ketogenic diet (KD), a low-carbohydrate, high-fat diet promoting ketone metabolism, confers cognitive benefits in aged C57BL/6 mice, and in the PS1/APP mouse model of early-onset AD. Here, we evaluated the effects of KD in a humanized APOE4 AD mouse model. KD significantly improved composite cognitive performance and spatial working memory, with pronounced effects in females. Synaptic plasticity, measured via long-term potentiation (LTP), was likewise enhanced exclusively in females. Transcriptomic and protein analyses revealed KD-induced activation of CREB pathway, marked by increased phosphorylation of ERK and CREB in female brains. Moreover, KD selectively reduced pro-inflammatory cytokine levels in females. These findings demonstrate sex-specific neuroprotective effects of KD in APOE4 mice and suggest its potential therapeutic role in mitigating AD risk in APOE4-positive women.

**Graphical Abstract:**

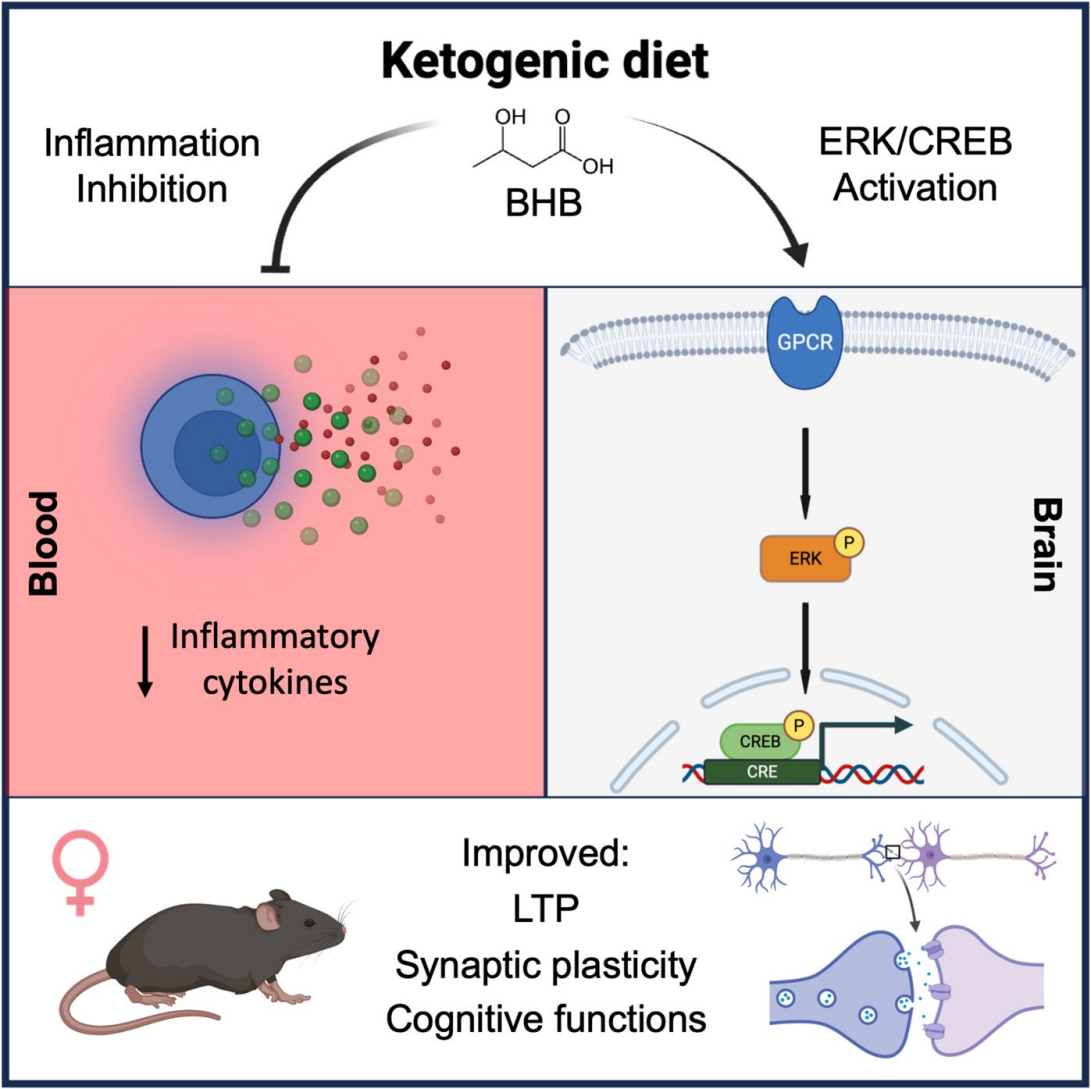

**Supplementary Information:**

The online version contains supplementary material available at 10.1007/s11357-025-01998-8.

## Introduction

Alzheimer’s disease (AD) is the leading cause of dementia in the developed world. Inheritance of ε4 allele of apolipoprotein E (APOE4) is the strongest genetic determinant of AD risk, with APOE4 females the most at risk [1, 2]. In the USA, about 20% of the population bear one APOE4 allele, most commonly the APOE3/E4 genotype. There are approximately 34 million APOE3/E4 females in the USA, at ~ fourfold increased AD risk at 70 years of age vs. E3/E3. APOE3/E4 males of the same age experience a smaller ~ threefold increase in AD risk [1–3].

Both glucose and lipid-derived ketone bodies support the bioenergetic health of the brain. Humans bearing APOE4 experience bioenergetic defects in cerebral glucose oxidation [4–8], and induce brain proteins responsible for ketone utilization [6]. The human APOE4 allele facilitates brain ketogenic metabolism in humans [7] and mice [9], long before development of mild cognitive impairment (MCI) or AD.

The ketogenic diet (KD) contains much higher levels of lipid and much lower levels of carbohydrate than a standard carbohydrate-rich control diet (CD) [10]. On the KD, the high levels of fat and low levels of carbohydrate increase fatty acid oxidation and ketogenesis, resulting in the production of β-hydroxybutyrate (BHB), a process known as ketogenesis. BHB can act as a signaling molecule with drug-like effects and serve as an alternative energy source to carbohydrates. The KD is widely used in both clinical and non-clinical settings, and it is considered the standard of care for childhood epilepsy refractory to anti-epileptic drugs [11].

We and others have previously shown that the KD protects C57BL/6 mice from age-related memory loss [10, 12]. We have also recently shown that the KD, and BHB itself, rescues deficits in synaptic plasticity as measured by long-term potentiation (LTP) in the PS1/APP mouse model of AD that recapitulates a genetically rare form of AD making up less than 1% of total AD cases [13]. In that study, the KD appeared to support increased LTP and synaptic plasticity through an increase in p-ERK and p-CREB pathways and of brain-derived neurotrophic factor (BDNF) [13]. We have also found that BHB increases microglial phagocytosis of beta-amyloid in vitro [14] and in vivo [15].

Here, we describe the effects of KD on cognition, LTP, synaptic plasticity pathways, and inflammation in mice bearing the humanized APOE4 allele.

## Methods

### Animals and diets

APOE4 [B6(SJL)-*Apoe*^tm1.1(APOE*4)Adiuj^*/*J] mice were purchased from Jackson Laboratories. Animals were bred in house, and both male and female mice were used for these studies. Mice were maintained on a 12-h light–dark cycle in a HEPA-filtered room where temperature (22–24 °C) and humidity (40–60%) were controlled. Water was provided ad libitum throughout the entire study, and health screens were performed on sentinel mice (housed on bedding from the study mice) every 3 months and tests included aerobic cultures and serology (MPV, MHV, MPV, MVM, M.pul, TMEV (GDVII), Ectro, EDIM, Sendai). All tests were negative throughout the study. At 10 months of age, animals were divided into two diet groups (control or ketogenic diet), counterbalanced by body weight, and individually housed to control food intake. Throughout the study, mice were fed isocaloric amounts (11.2 kcal/day) daily before the start of the dark cycle (~ 5 PM) and the mice consistently ate their daily food allotment. The CD consisted of (%kcal) 10% protein, 74% carbohydrate, and 16% fat, and the KD consisted of (%kcal) 10% protein, < 0.5% carbohydrate, and 89.5% fat (Supplementary Table 1). Diets were made in-house at least every 4 months and stored at 4 °C. All animal protocols were approved by the UC Davis Institutional Animal Care and Use Committee and conducted in accordance with the National Institutes of Health (NIH) Guide for the Care and Use of Laboratory Animals.

The starting age (10 months) for the diet groups was selected to represent middle age in C57BL/6 J mice and an age ~ equivalent to a 40-year-old human [16], which reflects a time when many people make diet changes for health reasons. The mice were then studied at 22 months of age to investigate the long-term effects of a KD on memory in aged APOE4 mice. This age was selected to represent old age in C57BL/6 J mice and an age ~ equivalent to a human in their 60 s [16].

After 12 months of dietary intervention, at 22 months of age, animals underwent behavior testing and body composition analysis. Mice were then exsanguinated under isoflurane anesthesia after an ~ 12-h fast. Tissues were collected, weighed, and snap frozen for gene and protein expression. Freshly isolated hippocampi were collected from a subset of animals for long-term potentiation assessment.

### Ketone measurement

Blood β-hydroxybutyrate level was measured using a Precision Xtra glucose and ketone monitoring system (Abbott, Chicago, IL, USA). Blood was obtained through tail nick in both the fed (3-h post-prandial) or fasted (approximately 12-h post-prandial) state.

### Body composition

At ~ 22 months of age, prior to euthanasia, body composition was evaluated using NMR relaxometry (EchoMRI-100 V; EchoMRI LLC, Houston, TX, USA).

### Mouse behavior tests

Mouse behavior tests were all conducted during the light cycle. Mice were allowed to acclimate to the test room for 30 min to minimize stress. Prior to testing, all equipment was cleaned with a 10% bleach solution followed by 70% ethanol, and 70% ethanol was used to clean between testing trials. A battery of motor and cognitive tests including (in order of testing) novel object recognition (NOR) test, Y-maze, grid wire hang, Barnes maze, and rotarod were used to evaluate the effect of dietary interventions on cognition and motor function.

### NOR test

The NOR test was conducted to assess recognition memory in mice. The day before NOR testing, mice underwent an acclimation session to habituate them to the environment. For this acclimation, animals were placed in the arena (40 × 40 × 40 cm white acrylic box) and were allowed to freely explore the arena for 15 min. The next morning, two identical objects were placed in the arena, and the mouse was allowed to explore the objects for 10 min. One hour after the morning session, one of the identical objects from the morning session was replaced (side randomized) with a novel object, then mice were allowed to explore the objects for 10 min. Videos were recorded with an overhead camera, and the movement of the mouse was tracked using the EthoVision XT17 (Noldus; Wageningen, the Netherlands). A mouse was considered exploring an object if the nose of the mouse was pointed toward the object and was within 2 cm of the object. Percent (%) time exploring novel objects was calculated using time spent (seconds) exploring objects as follows:

$$(\mathrm{novel}/(\mathrm{novel}\:+\:\mathrm{identical}))\:\times\:100\%$$

### Y-maze spontaneous alternation test

The Y-maze spontaneous alternation test was conducted to assess spatial working memory in mice. A Y-shaped maze where each arm was 120° from each other and 35 × 8 × 15 cm (*L* × *H* × *W*, white acrylic) in dimensions was used for this test. After being placed in the center of the maze, mice were allowed to move around the maze for 6 min. An overhead camera was used to record videos, and the movement of the mice was tracked using the EthoVision XT17 software. An arm entry was counted when the center point of the mouse traveled to the distal side of the arm (more than one-third of the arm length) and returned to the center of the maze. Non-repeating triplets were defined as when the mouse enters all three different arms consecutively. The percentage (%) alternation was calculated as:

$$((\#\;\mathrm{non}-\mathrm{repeating}\;\mathrm{triplets})/(\#\;\mathrm{total}\;\mathrm{arm}\;\mathrm{entries}\:-\:2))\:\times\:100\%$$

### Grid wire hang test

The grid wire hang test was conducted to assess isometric muscle endurance. A stainless-steel wire mesh screen with 1 × 1 cm grids and 1-mm wires supported on a plastic box (40 cm tall) was used for this test. Towels were placed at the bottom of the box to cushion the mice during falls. Mice were placed on the top of the screen, and the screen was gently shaken to ensure a firm grip. The screen was then inverted, and time to fall was recorded. All animals were tested twice with an intertrial interval of ~ 30 min. Maximal hanging impulse was calculated as:

$$\mathrm{maximum}\;\mathrm{hanging}\;\mathrm{time}\;(\mathrm s)\:\times\:\mathrm{body}\;\mathrm{weight}\;(\mathrm{kg})\:\times\:9.8\;\mathrm N\;\mathrm{kg}^{-1}$$

### Barnes maze

The Barnes maze test was conducted to measure spatial learning and memory in mice. On the first three testing days, mice underwent 1, 2, or 3 training trials per day with an intertrial interval of 15–30 min, followed by a probe trial on day 4. The maze consisted of a 92-cm white acrylic circular disk with 20 5-cm holes evenly distributed on the periphery and was elevated ~ 75 cm above the ground. An overhead LED light source was used to illuminate the maze, and signs were placed around the maze to be used as visual cues. A black escape box equipped with a step and fresh bedding was secured under the target hole as a shelter for the mice. For the training trials, mice were placed under an opaque bucket in the middle of the maze. After ~ 10 s, the light was turned on, the bucket was removed, and the mouse was allowed to explore the maze for 3 min or until they entered the escape box though the target hole. If the mice did not enter the escape box within 3 min, they were directed to the target hole and allowed to rest for at least 30 s. On the probe day, the escape box was removed, and mice were allowed to explore for 2.5 min. Videos were recorded with an overhead camera, and latency to target (s), pathlength to target (cm), and % time in target quadrant (s) were calculated using EthoVision XT17 software (Noldus; Wageningen, the Netherlands).

### Rotarod

The rotarod test assessing balance and motor function deficits was conducted on a Rota Rod Rotamex (Columbus Instruments). Clean towels were placed at the bottom of each lane to cushion the mice during falls from the rod. Mice were placed on the rod at 4 rpm which then accelerated at 1 rpm/6 s to a maximum of 40 rpm. Three trials were conducted each day for 2 days with an intertrial interval of at least 20 min. The latency to fall was recorded.

### Induction of hippocampal long-term potentiation by high-frequency stimulation

The preparation of mouse hippocampal slices and the induction of hippocampal long-term potentiation (hLTP) by high-frequency stimulation of the Schaffer collateral afferents were conducted as followed. Coronal slices (300 µm) of mouse hippocampus were prepared from APOE4 mice of CD and KD. The animals were subjected to deep anesthesia with isoflurane and decapitated. The brain was rapidly removed and transferred to a modified artificial cerebrospinal fluid (HI-ACSF) containing (in mM) 220 sucrose, 2 KCl, 0.2 CaCl_2_, 6 MgSO_2_, 26 NaHCO_3_, 1.3 NaH_2_PO_4_, and 10 d-glucose (pH 7.4, set by aeration with 95% O_2_ and 5% CO_2_). Coronal brain slices were cut in ice-cold modified artificial cerebrospinal fluid (ACSF) with the use of a DTK-1000 D.S.K. Microslicer (TedPella, Inc., Redding, CA, USA). The slices were immediately transferred into an ACSF solution containing (in mM) 126 NaCl, 3 KCl, 2 CaCl_2_, 1 MgCl_2_, 26 NaHCO_3_, 1.25 NaH_2_PO_4_, and 10 d-glucose (pH 7.4, set by aeration with 95% O_2_ and 5% CO_2_) for at least 40 min at controlled temperature of 35 °C. After subsequent incubation for at least 1 h at room temperature, hemi-slices were transferred to the recording chamber, which was perfused with standard ACSF at a constant flow rate of ~ 2 ml/min. Recordings of field excitatory postsynaptic potentials (fEPSPs) were obtained from the stratum radiatum of the CA1 region of the hippocampus after stimulation of the Schaffer collateral afferents. Extracellular recording electrodes were prepared from borosilicate capillaries with an outer diameter of 1.5 mm (Sutter Instruments) and were filled with 3 mM NaCl (resistance, 1–2 MΩ). Baseline stimulation rate was 0.05 Hz. FEPSPs were filtered at 2 kHz and digitized at 10 kHz with a MultiClamp 700B amplifier (Molecular Devices, Sunnyvale, CA, USA). Data was collected and analyzed with pClamp 10.3 software (Molecular Devices). Slope values of fEPSPs were considered for quantitation of the responses. After 10 min of stable baseline recording of fEPSPs evoked every 20 s by application of a constant current pulse of 0.2–0.4 mA with a duration of 60 ms at the current intensity set to evoke 50–60% of the maximal response, LTP was elicited by high-frequency stimulation (HFS), consisting of two trains of 100-Hz (1-s) stimulation with the same intensity and pulse duration used in sampling of baseline fEPSPs. Recording was then continued for 60 min with stimulation of fEPSPs every 20 s.

### RNA sequencing and gene ontology by ingenuity pathway analysis

RNA was isolated from 10 mg of frozen murine brain cortex (*N* = 4–6 per group; 22-month-old APOE4 males/females on control or ketogenic diet), using the RNeasy Mini Kit (QIAGEN) according to the manufacturer’s instructions. Extracted RNA was used for NGS library preparation following Illumina’s instruction (TruSeq Stranded Total RNA Library Prep Gold), while sequencing was performed on NovaSeq 6000 (Illumina) running in a 50-bp paired-end mode.

Quality control of raw sequences was performed using FASTQC (Babraham Institute). Samples were then aligned to the murine reference genome (*Mus musculus*; UCSC, mm10), using STAR. Mapped sequences were processed with HTSeq software, integrated into STAR, to count reads per gene as a first raw measure of gene expression levels. Samples were scaled using upper-quartile normalization while the unwanted variation was estimated through RUVg function from RUVSeq R package [17].

Differential expression analysis was conducted using the negative binomial GLM approach implemented in edgeR, and Benjamini–Hochberg procedure was used to adjust *p* value to obtain the false discovery rate (FDR).

All differentially expressed genes (DEGs) with FDR ≤ 0.1 and absolute fold change (FC) ≥ 1.5 were used as input for ingenuity pathway analysis (IPA) (QIAGEN), in order to identify canonical pathways and upstream regulators involved. All RNA-sequencing (RNA-seq) results are plotted using ggplot2 R package.

### Tissue homogenate preparation and western blot analysis

Hippocampi were homogenized in lysis buffer (150 mM NaCl, 10 mM NaH_2_PO_4_, 1 mM EDTA, 1% Triton X-100, 0.5% SDS) with protease inhibitor cocktail and phosphatase inhibitor (Sigma). Equivalent amounts of protein were analyzed by 4–20% Tris–Glycine gel electrophoresis (Invitrogen). Proteins were transferred to polyvinylidene difluoride membranes and probed with antibodies. Visualization was enabled using enhanced chemiluminescence (GE Healthcare Pharmacia). The following primary antibodies (dilutions) were used: phospho-CREB (1:1000, Cell Signaling, #9198), CREB (1:1000, Cell Signaling, #9197), phospho-ERK (1:1000, Cell Signaling, #9106), ERK (1:1000, Cell Signaling, #9102), and β-actin (1:5000, Cell Signaling, #3700). Secondary antibodies were HRP-conjugated anti-rabbit or anti-mouse antibody (1:1000, Cell Signaling, #7074 and #7076).

### Plasma analysis

At termination, blood was collected from cardiac puncture in EDTA tubes (Becton Dickinson; Franklin Lakes, NJ, USA). Plasma was isolated by centrifuging blood at 2000 × *g* for 15 min at 4 °C and sent to the UC Davis Nutrition Department for analysis. Enzymatic colorimetric assays were completed using kits according to the manufacturer’s instructions: free fatty acids (FujiFilm Wako Diagnostics, Mountain View, CA, USA), triglycerides, and total cholesterol (Fisher Diagnostics, Middletown, VA, USA). LDL and VLDL were precipitated using reagents from Abcam (Cambridge, MA, USA), and supernatant HDL-C was measured (Fisher Diagnostics, Middletown, VA, USA).

Concentrations of plasma cytokines and chemokines (IL-12p40, IL-12p70, IL-17, IL-1α, IL-1β, IL-2, CCL3, CCL4, CCL5, IL-6, TNF-α, GM-CSF, IFN-γ) were detected using Millipore’s MILLIPLEX Mouse Cytokine/Chemokine Magnetic Bead Panel as previously described [18]. Twenty-five microliters of plasma diluted 1:2 (run in duplicate) was incubated with capture antibody-coated beads in a 96-well filter-bottom plate overnight at 4 °C while shaking. After incubation, the plate was washed twice. Detection antibodies were then added and incubated for 1 h at room temperature with shaking. The reaction mixture was detected by the addition of streptavidin–phycoerythrin and incubated on a plate shaker at room temperature for 30 min. Following two washes, beads were resuspended in sheath fluid for 5 min on the plate shaker. The plate was then read on a Bio-Plex 200 system (Bio-Rad Laboratories; Hercules, CA, USA) and analyzed using Bio-Plex Manager software (Bio-Rad Laboratories).

### Statistical analysis

Statistics were performed using GraphPad Prism 10 (GraphPad Software Inc., San Diego, CA, USA). Descriptive statistics are expressed as mean ± standard error of the mean (SEM). Outliers within each group were identified using the ROUT method, a robust nonlinear regression-based method for evaluating the influence of outliers [19], and excluded from statistical analysis. For NOR test, animals with a total exploration time (novel + identical) < 3 s were excluded. The *z*-score for each domain cognitive test was calculated as the number of standard deviations from the mean of the reference group (combined male and female CD, male CD, and female CD), and a composite score was then computed by averaging the *z*-scores from the select domain tests to avoid redundant tests. The cognition score included % time exploring the novel object in the novel object recognition test, % alternation in the Y-maze, and latency to the target hole in the Barnes maze probe trial. The directionality of scores were adjusted, and a higher positive score indicated better performance. Prior to statistical inference tests, the analysis of residuals was performed to validate the normality and homoscedasticity assumptions using QQ and residual plots as well as D’Agostino and Pearson test for normality. For behavior analyses, two-way ANOVA was used to evaluate the overall main effects of sex and diet and its sex-by-diet interaction. Sex-specific group comparisons between diets were conducted using unequal variances (Welch’s *t*-test or Mann–Whitney test) if data were not normally distributed. In addition, the Benjamini–Hochberg’s FDR method was applied to control for multiple testing with an FDR < 0.1 being considered significant. For serum cytokine analysis, missing values of plasma cytokines below the limit of detection (LOD) were imputed as one-half of the marker-specific minimum of the detectable values prior to statistical analysis. Individual cytokine *z*-scores were calculated as the number of standard deviations from the overall mean of the pooled sample for each cytokine. The composite *z*-score was then computed by averaging the *z*-scores across pro-inflammatory cytokines (IL-12p40, IL-12p70, IL-17, IL-1α, IL-1β, IL-2, CCL3, CCL4, CCL5, IL-6, TNF-α, GM-CSF, IFN-γ). Two-way ANOVA was used to evaluate the overall main effects of sex and diet and its sex-by-diet interaction followed by Fisher’s least significant difference (LSD) post hoc tests for pairwise comparisons. For individual cytokine analysis, if data were not normally distributed, the data was log-transformed prior to statistical tests or analyzed using aligned ranks transformation ANOVA (IL-1β) as appropriate. For lipid analysis, the Bonferroni correction was used to adjust for multiple comparisons. For LTP and western blot, comparison of the mean values was performed using unpaired Student’s *t*-test, or two-way ANOVA with Tukey’s post hoc tests. Exact sample sizes and statistical test used for each comparison were provided in corresponding figure legends. Statistical significance was determined at two-sided *p* < 0.05.

## Results

### KD increases circulating BHB in APOE4 mice

**Fig. 1:**
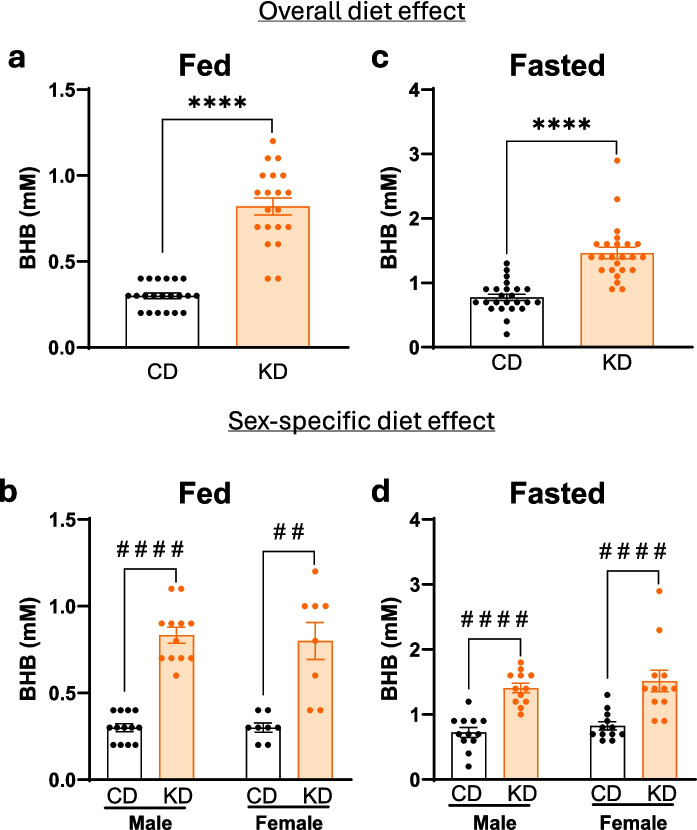
Ketogenic diet increases circulating β-hydroxybutyrate. Blood β-hydroxybutyrate (BHB) was measured in both male and female APOE4 mice fed a control diet (CD) or ketogenic diet (KD) in the fed (**a** and **c**, measured 3 h after the daily feeding) and fasted (**b** and **d**, immediately prior to meal period). Two-way ANOVA shows significant differences between diet groups, adjusted for sex, in the fed (*F*(1, 18) = 151.1) and fasted (*F*(1, 44) = 45.12) state, ****p < 0.0001. No significant sex difference and sex–diet interaction were found in the fed or fasted state. Data are presented as mean ± SEM, *n* = 8–13 per group. ^##^*p* < 0.01 and ^####^*p* < 0.0001 indicate significant differences between diets based on unequal variances Welch’s *t*-test or Mann–Whitney test

Circulating BHB was measured to assess the level of ketosis with the KD vs. the CD. The KD elevated circulating BHB levels by approximately threefold in fed mice and twofold in fasted mice vs. CD (Fig. 1a, c). While mean BHB levels were not significantly different between KD males and females in fed or fasted state, variability in female BHB levels was greater than in males (Fig. 1b, d). Although all groups were fed isocaloric quantities, APOE4 mice fed KD for 12 months weighed significantly more than CD-fed animals (Supplementary Fig. 1a) and had an overall increase in fat mass (Supplementary Fig. 1c) and lean mass (Supplementary Fig. 1e). The increase in fat appears to be the primary contributor to the increase in body weight (Supplementary Fig. 1a–f). Although there were significant diet-related changes in body composition and body weight, the magnitude of these changes was small, and the body weights were at typical levels for aged mice.

### KD increases total and high-density lipoprotein cholesterol levels

Plasma triglyceride, free fatty acid (FFA), total cholesterol, and high-density lipoprotein (HDL), and non-HDL (low- and very low-density lipoprotein) cholesterol levels were measured in fasted blood samples from APOE4 mice after 12 months on diet (Supplementary Table 2). Females had significantly lower total and HDL cholesterol level compared to males (sex main effect, *p* < 0.05). There was also a diet main effect involving total and HDL cholesterol (*p* < 0.001) with an increase in KD-fed mice. In particular, male animals fed KD were found to have significantly higher total and HDL cholesterol levels (*p* < 0.05) compared to CD. Females on KD showed a similar trend for an increase in total and HDL cholesterol, but this was not significant (*p* = 0.08). Furthermore, as the ketogenic diet has been shown to be beneficial to liver health and to reverse non-alcoholic fatty liver disease [20], liver weights were measured. We found that the KD had no effect on liver weight in either sex (Supplementary Fig. 1 g, h).

### KD increases muscle mass and muscle endurance

As the KD has been shown to preserve muscle function and mass in old age [10], representative muscles from the lower hindlimb were weighed. KD-fed APOE4 mice had relatively larger gastrocnemius muscles, compared to CD-fed mice (Supplementary Fig. 1i). When sexes were evaluated separately, male KD mice trended to have larger gastrocnemius muscles (male KD vs. CD *p* = 0.0635; Supplementary Fig. 1j). However, soleus muscle weight showed no difference between groups (Supplementary Fig. 1 k, l). The grid wire hang and rotarod tests were used to determine isometric muscle endurance and motor coordination in 22-month-old APOE4 mice. Overall, mice fed KD had improved performance compared to the CD in the wire grid hang test (Supplementary Fig. 2a). There was a significant improvement in isometric muscle endurance in male mice fed KD compared to CD, but this measure did not reach a significant difference between the two diet groups in female mice (Supplementary Fig. 2b). Rotarod performance was not significantly different between the diet groups when evaluated overall, by diet, or by sex (Supplementary Fig. 2c, d).

### KD improves composite cognitive score and significantly enhances spatial working memory in APOE4 females compared to CD

**Fig. 2:**
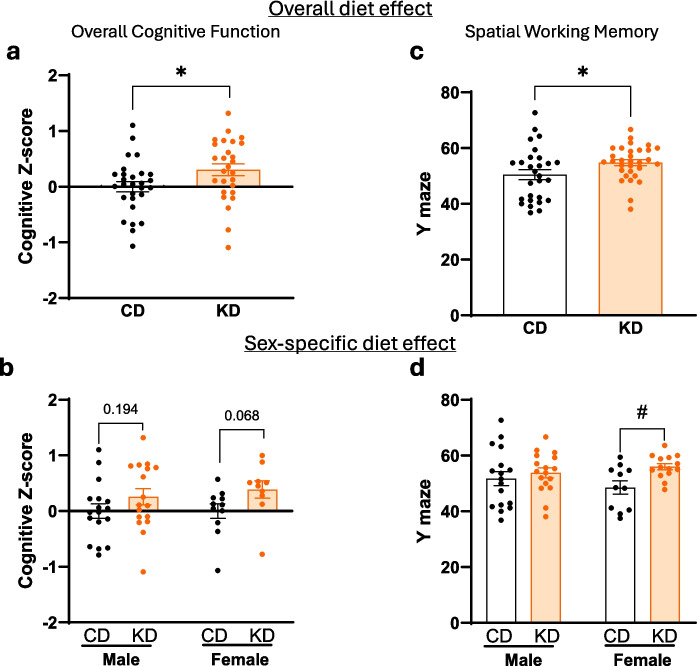
Ketogenic diet improves overall cognition and spatial memory. **a**, **b** Composite *z*-score of three domain cognitive tests (Y-maze (% alterations), Barnes maze (latency), and NOR (% time exploring novel object) was determined in male and female APOE4 mice fed a KD, normalized to the mean of the control diet. Two-way ANOVA shows a significant overall diet effect, adjusted for sex, *F*(1, 51) = 4.836, *p < 0.05). **c**, **d** Percent (%) of alternations in the Y-maze spontaneous alternation test showed a significant overall diet effect in % alternations adjusted for sex (*F*(1, 55) = 5.113, *p < 0.05). No significant sex difference or sex–diet interaction were found. Data are presented as mean ± SEM, *n* = 10–17 per group. ^#^*p* < 0.05 indicates significant differences between diets based on unequal variances Welch’s *t*-test (FDR < 0.1)

The overall effect of the KD on memory in 22-month-old APOE4 mice was measured with a composite cognitive score that combined cognitive measures of multiple discrete domains and was calculated as the mean of three domain *z*-scores (Barnes maze latency, Y-maze % alternating triplets, and NOR % time exploring novel objects) to yield an overall score. KD-fed APOE4 mice of both sexes had significantly higher composite *z*-scores compared to CD in APOE4 mice indicating a cognitive improvement (Fig. 2a; *p* < 0.05). When separated by sex, we observed the same trends in both APOE4 KD-fed females and males, although not statistically significant (Fig. 2b; female *p* = 0.068 and male *p* = 0.194). When each domain test was studied individually, KD-fed APOE4 mice showed a higher percentage overall of alternating triplets in the Y-maze compared to CD (Fig. 2c), indicating improved spatial working memory. However, when broken down by sex, the significant improvement in spatial working memory was only observed in female APOE4 mice fed KD vs. CD (*p* < 0.05), not in males (Fig. 2d; *p* = 0.44). There were no significant differences in NOR tests (Supplementary Fig. 3a, b) and in Barnes maze testing outcomes (Supplementary Fig. 3c, d) between the two diets in APOE4 mice.

### KD rescues LTP in female APOE4 mice more than in males

**Fig. 3:**
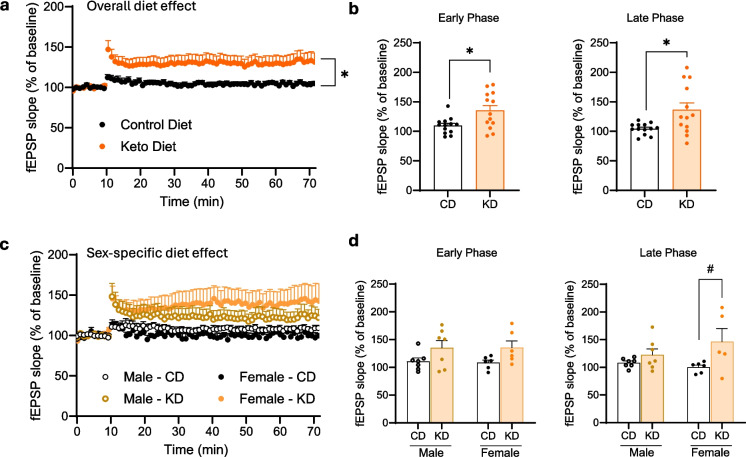
Ketogenic diet rescues LTP in APOE4 mice sex-specifically. **a**, **b** Time course scatter plot and bar graph of early and late phases of hLTP induced with high-frequency stimulation showing that ketogenic diet increases the amplitude of both early and late phases of LTP in APOE4 mice. **c**, **d** Time course scatter plot and bar graph of early and late phases of hLTP induced with high-frequency stimulation divided by sex. Data show that a ketogenic diet increases the amplitude of late phase of LTP in APOE4 female mice. Data were compiled from recordings using slices obtained from the groups of male/CD (*n* = 7), male/KD (*n* = 7), female/CD (*n* = 6), and female/KD (*n* = 6), and are presented as mean percent change in fEPSP slope ± SEM from baseline. Statistical analysis was performed by Student’s *t*-test and two-way ANOVA followed by Tukey’s post hoc tests. *p < 0.05; ^#^*p* < 0.05 within sex group

LTP is thought to be a primary marker of synaptic plasticity, which is the neurophysiological basis of memory [21]. It was previously shown that expression of humanized APOE4 decreases LTP in 20-month-old mice [22]. Similarly, the 22-month-old APOE4 mice in our study on the CD had minimal fEPSP slope, around 110% of baseline (Fig. 3a, b). By contrast, age-matched APOE4 mice consuming KD for 12 months experienced significantly higher early- and late-phase LTP of around 140% of baseline (Fig. 3a, b). We then analyzed the data by sex, observing that only female APOE4 mice had a significant increase in the late phase of LTP when fed KD vs. CD (Fig. 3c, d). Although male LTP trended higher on KD, the difference was not significant (Fig. 3c, d).

### Gene expression analysis of brain cortex of APOE4 mice reveals that KD enhances CREB synaptic plasticity pathway

RNA-seq analyses were carried out on brain cortex samples of male and female APOE4 mice, with at least four biological replicates for each condition, taking into account sex and diet regimen. We compared the transcriptomic profiles of APOE4 mice fed KD vs. CD, using a significance cutoff of FDR ≤ 0.1 and an absolute FC ≥ 1.5. The assessment of KD-regulated gene expression revealed 871 DEGs in females (573 up-regulated and 298 down-regulated) and 257 DEGs in males (183 up-regulated and 74 down-regulated) (Supplementary Table 3).

**Fig. 4:**
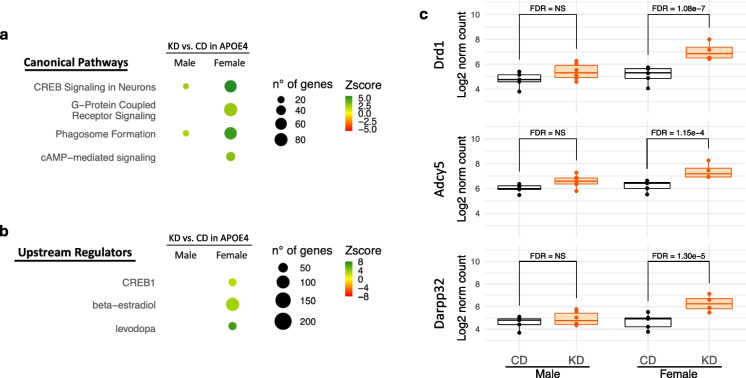
Ketogenic diet improves CREB signaling pathway. Canonical pathways (**a**) and upstream regulators (**b**) were obtained by ingenuity pathway analysis of differentially expressed genes associated with ketogenic diet effects in APOE4 mouse brain. Terms with *p* value < 0.05 are reported. Colors are related to degrees of *z*-scores while dimensions of dots reflect the number of genes involved. **c** Quantitative analysis of Drd1, Adcy5, and Darpp32 expression in CD- and KD-fed male (left) and female (right) mice is shown. A false discovery rate (FDR) is reported in a plot when significant at FDR ≤ 0.1; otherwise, NS indicates non-significant

To identify which cellular processes are associated with the identified DEGs, these gene datasets were subject to IPA. Considering *z*-score greater than 2 or less than − 2 for significant activation or inhibition status in the pathway analysis, multiple canonical pathways were identified as significant (Fig. 4a). The KD activated several pathways known to be relevant to neuronal functions, especially in female mice. Among them, CREB (cAMP response element-binding protein) pathway was the primary process enhanced by the KD, encompassing CREB signaling in neurons, G-protein-coupled receptor signaling, and cAMP-mediated signaling. Furthermore, IPA identified the upstream regulators, i.e., the factors involved in the control of the expression of altered genes. CREB1, estradiol, and l-DOPA emerged as the most likely (*z*-score > 2) mediators of the transcriptional remodeling triggered by KD in females (Fig. 4b). In addition, several genes known to activate the synaptic plasticity factor CREB were significantly induced by KD in female but not in male APOE4 brains (Fig. 4c). These include dopamine receptor D1 (Drd1), adenylate cyclase 5 (Adcy5), and dopamine and cAMP-regulated phosphoprotein 32 kDa (Darpp32, also known as protein phosphatase 1 regulatory inhibitor subunit 1B, Ppp1r1b).

Another transcriptomic pathway enhanced sex-specifically by KD was phagosome formation, which was increased more in females than in males (Fig. 4a). Microglial phagocytosis of beta-amyloid is thought to facilitate its clearance and reduce its toxicity [23].

### KD significantly induces p-CREB and p-ERK biochemical pathways, thought to underlie synaptic plasticity, only in female APOE4 mice

**Fig. 5:**
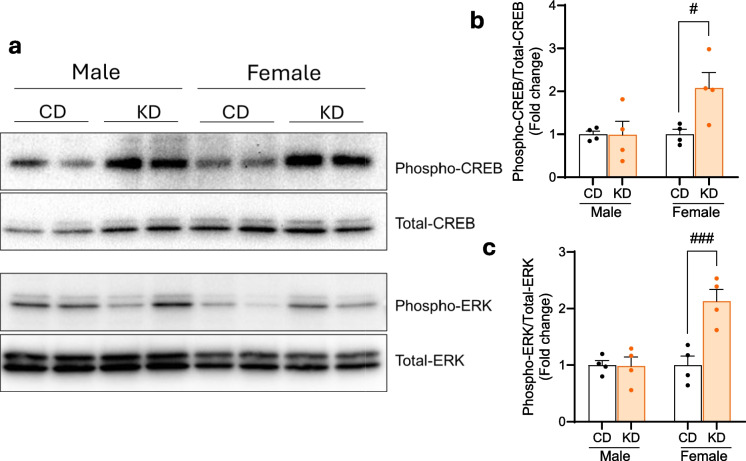
Ketogenic diet’s biochemical effect is significant in female hippocampi. **a** Representative western blot and **b**, **c** quantification of phospho-CREB and phospho-ERK. Ketogenic diet increases the expression of phospho-CREB and phospho-ERK in female APOE4 mice. Data are presented as means ± SEM. *n* = 4 in all group. Statistical analysis was performed by two-way ANOVA followed by Tukey’s post hoc tests. ^#^*p* < 0.05; ^###^*p* < 0.001

As transcriptomics pointed toward synaptic plasticity pathways involving CREB, the activities of CREB and ERK proteins were also investigated. Hippocampal western blots demonstrated significantly increased phosphorylation of both CREB and ERK only in KD-fed APOE4 females, not males, vs. their CD counterparts (Fig. 5).

### KD significantly reduces circulating inflammatory cytokines vs. CD in APOE4 females only

**Fig. 6:**
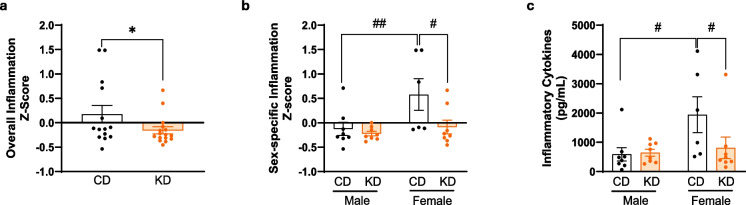
Ketogenic diet reduces circulating inflammatory cytokines in female APOE4 mice. **a**, **b** Composite *z*-score of 13 pro-inflammatory cytokines (IL-17, IFN-γ, TNF-α, CCL3, CCL4, CCL5, IL-2, IL-12p70, IL-12p40, IL-6, IL-1α, IL-1β, GM-CSF) was determined in male and female APOE4 mice fed CD or KD, and the data were normalized to the global *z*-score. Two-way ANOVA shows a significant overall diet effect (*F*(1, 26) = 5.174, *p < 0.05). **c** Sum of pro-inflammatory cytokines showed a significant overall sex effect (*F*(1, 26) = 4.881, *p* < 0.05). Data is presented as mean ± SEM, *n* = 6–8 per group. ^#^*p* < 0.05 and ^##^*p* < 0.01 indicate significant differences between groups based on Mann–Whitney test or uncorrected Fisher’s LSD

Circulating pro-inflammatory cytokines were assessed in plasma samples from APOE4 mice after 12 months on CD or KD, and composite inflammation score of 13 pro-inflammatory cytokines was calculated (Fig. 6a, b). Overall, there were both sex (*p* < 0.05) and diet (*p* < 0.05) effects on systemic inflammation. On CD, APOE4 females had a significantly higher inflammation score compared to APOE4 males (*p* < 0.01), which was significantly decreased (*p* < 0.05) when fed KD (Fig. 6b). The concentrations of each of the 13 cytokines were summed in Fig. 6c. Females on CD had a significantly higher summed concentration of cytokines than males on CD by around threefold. KD significantly reduced the female sum of cytokines compared to CD about twofold. When assessed individually, KD significantly lowered 9 of 13 circulating systemic cytokines in females compared to CD (Supplementary Fig. 4).

## Discussion

APOE4 is the strongest genetic risk factor for Alzheimer’s disease and has a disproportionately greater impact in women. APOE4 inheritance increases AD risk fourfold by age 70 in females relative to non-carrier females, while the increase in AD risk in E4-bearing males is threefold [1, 2]. The allele frequency of APOE4 is about 20%; thus, in the US population of approximately 340 million, there are around 68 million individuals carrying at least one APOE4 allele, putting around 34 million females at increased risk of AD. In addition, the presence of an APOE4 allele increases the chance of experiencing an adverse event to modern antibody-directed beta-amyloid lowering strategies (e.g., Leqembi) [24–27]. Thus, women bearing APOE4 are the population at highest risk of AD and could specifically benefit from novel dietary and therapeutic strategies.

Furthermore, humans bearing APOE4 experience a brain bioenergetic defect long before the onset of dementia. Both glucose and lipid-derived ketone bodies support the bioenergetic health of the brain [28]. In this work, we tested the hypothesis that providing APOE4 mice with alternative metabolic support through KD, which induces endogenous BHB production, would ameliorate their cognitive and memory deficits.

Long before the age of onset of MCI or AD, young adult APOE4 carriers (~ 32 years) show impaired cerebral glucose uptake and utilization [4–6, 8], and upregulation of glucose transporters [7]. As a consequence of APOE4 inheritance, these individuals experience a significant increase in the BHB-metabolizing proteins SCOT, AACS, and BHB transporter protein MCT2 in their brains [7]. Similar to humans, APOE4 female mice produce and metabolize more ketones than their APOE3 counterparts [9]. These results in humans and mice are consistent with the hypothesis that inheritance of APOE4, because it is known to carry less lipid than APOE3, may set up a bioenergetic deficit that can be overcome by KD-mediated lipid supplementation.

**Fig. 7:**
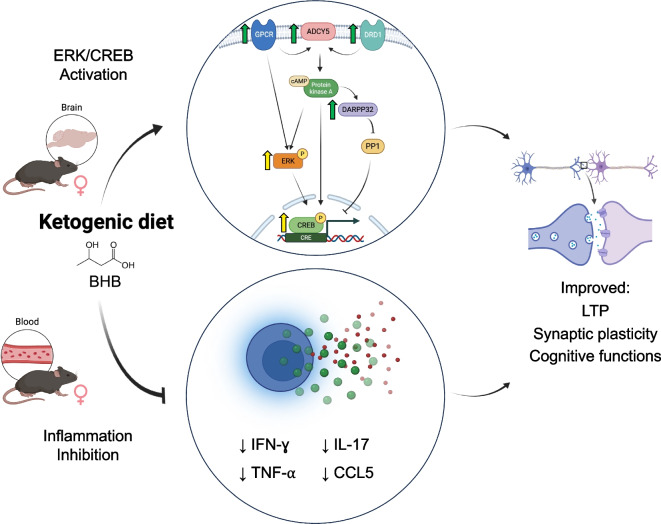
Ketogenic diet enhances LTP, synaptic plasticity, and cognitive functions in female APOE4 mice via systemic inflammation reduction and CREB-dependent gene expression remodeling. Schematic representation of the dual mechanisms by which long-term KD enhances cognitive functions in female APOE4 mice. At the systemic level, KD reduces circulating pro-inflammatory cytokines (e.g., IFN-γ, TNF-α, IL-17, CCL5), leading to decreased peripheral inflammation. In parallel, KD-induced activation of G-protein-coupled receptors (e.g., Drd1, Gpr52, Gpr83) stimulates ERK-CREB signaling in the brain, triggering transcriptional remodeling of genes involved in synaptic plasticity. Together, these pathways converge to improve synaptic function and cognitive performance. Green and yellow arrows indicate increases in RNA and phosphoprotein levels, respectively

We found that KD rescues multiple memory deficits in APOE4 mice. Feeding KD for 12 months significantly rescued overall cognitive functions measured by composite score, spatial working memory, and LTP outcomes in APOE4 mice when both sexes were combined. When sexes were separated, spatial working memory and LTP were significantly improved in female APOE4 mice only (Figs. 2 and 3). There was no significant difference in fed or fasting BHB levels between females and males (Fig. 1), suggesting that any female-specific benefit was not the result of higher BHB level in females. In addition, KD affected fivefold more CREB-related genes in females than in males (69 vs. 14 genes) (Fig. 4a). Female APOE4 mice had over fourfold more phagosome pathway–associated genes affected than males (71 vs. 16 genes) (Fig. 4a). KD significantly induced individual upstream regulators of CREB Drd1, Adcy5, and Darpp32, but only in females (Fig. 4c). Consistent with these transcriptional results, KD induced the biochemical synaptic plasticity pathway enzymes p-CREB and p-ERK in the hippocampi of female APOE4 mice only (Fig. 5). Based on these findings, we propose two potential mechanisms for KD’s female-specific rescue of spatial working memory and LTP (Fig. 7).

First, APOE4 inheritance increases inflammation that inhibits memory, and KD reduces that inflammation, preserving memory. It has been proposed that increased inflammation through complement system and microglia may reduce synaptic plasticity and memory in multiple mouse models of AD [29]. APOE4 mice have impaired memory on CD [30–32] and increased inflammatory poise of the innate immune system that includes macrophages and microglia [33]. In addition to the general increase in innate immune system activation conferred by APOE4, female APOE4 mice on CD have increased inflammation of primary microglia, astrocytes, and neutrophils vs. males [34–36], and a correlated pro-inflammatory status of peripheral and brain cytokines [37]. In humans, likewise, APOE4 inheritance increases the inflammatory tone of innate immune system cells, resulting in elevated levels of pro-inflammatory cytokines in the bloodstream [38, 39], and microglial activation in the brain as measured by Iba1 staining [40]. Thus, APOE4 confers an increase in the inflammatory tone of the innate immune system, with increased circulating inflammatory cytokines, and increased microglial inflammation in the brains of APOE4 humans [38–40] and mice [33, 34, 36].

Consistent with previous findings in APOE4 mice [33], we show significantly increased peripheral cytokines in female APOE4 mice on CD (Fig. 6b, c). KD significantly reduced inflammatory status in aggregate across sexes vs. CD (Fig. 6a), specifically lowering 9 of 13 circulating cytokines in female mice (Supplementary Fig. 4). We have previously demonstrated a dose-dependent anti-inflammatory effect of KD (both intermittent and continuous KD) on peripheral inflammatory cytokines in aged C57BL/6 mice vs. CD.

We previously showed that the KD’s suspected active principle, BHB, is dose-dependently anti-inflammatory to beta-amyloid-inflamed human-induced pluripotent stem cell (iPSC)-derived microglia, most potently at the 1 mM BHB concentration that is physiologically reached on KD but not on CD [13]. BHB injected intraperitoneally reduced Iba1-positive microglia, microglia-derived inflammatory cytokines, and microglial inflammasome formation in the brains of 5XFAD mice [15]. Similarly, KD fed to PS1/APP mice significantly reduced Iba1, CD11b, CD68, and Dectin-1 microglial inflammation markers [13]. Thus, it is possible that the KD, by suppressing inflammation peripherally and in the CNS, improves memory outcomes [41].

APOE4 inheritance causes a general increase in inflammatory tone in humans [38–40] and mice [33, 34, 36], and we observed a significantly higher level of 7 of 13 inflammatory cytokines in female vs. male mice (Supplementary Fig. 4). Colton’s group has shown that APOE4 inheritance not only increases innate immune inflammation (including macrophages and microglia) vs. APOE3 [33], but also suppresses the anti-inflammatory effect of beta-estradiol [29]. Thus, our observation of a female-specific elevation in pro-inflammatory cytokine levels on CD, compared to males, could result from estrogen’s reduced anti-inflammatory potency in the female APOE4 context.

Beta-estradiol levels decline in women after menopause. Analogously, beta-estradiol falls in aging female mice that lose estrous cycling [42]. If the results observed in APOE4 mice were to translate to humans, postmenopausal APOE4 women would face an increased risk of inflammation for at least two reasons: the natural drop in beta-estradiol levels after menopause, and an APOE4-dependent reduction in response to beta-estradiol’s anti-inflammatory effects.

The second potential mechanism involves KD-induced activation of the CREB synaptic plasticity pathway, specifically in APOE4 females. CREB is an important supporter of synaptic plasticity and memory [43] and disruption of CREB causes neurodegeneration [44]. Others have shown that CREB is deficient in APOE4 female mice [30, 31], and these animals have more spatial learning and memory deficits than males on CD [32]. Here we show that KD induced more CREB pathway–related genes in female than in male APOE4 brains (Fig. 4). Thus, a KD- and female-specific rescue of CREB signaling could explain the increase in synaptic plasticity–dependent endpoints LTP (Fig. 3d) and spatial working memory (Fig. 2d), uniquely in female APOE4 mice. A speculative possibility that unifies the two mechanisms above is that the female-specific rise in inflammation may cause decreased CREB in APOE4 females; however, further experiments are needed to confirm this hypothesis.

Alternatively, APOE4 inheritance shifts metabolism toward an increased utilization of ketone bodies, which are insufficiently produced while following a standard carbohydrate-rich diet. Indeed, APOE4 presence redirects substrate utilization from more insulin- and carbohydrate-dependent to more lipid- and ketone-dependent in mice [9]. Shang et al. showed that female APOE3 and APOE4 mice have higher plasma ketones than males at 6 months, as if females are more reliant on ketones as a metabolic substrate than males, and aged APOE4 females had approximately twice the circulating ketone levels compared to APOE3 females and both APOE3 and APOE4 males [9]. Consistent with this, APOE4 inheritance resulted in a lower circulating insulin level at 16 months and significantly lower blood glucose at 6 months, supporting the hypothesis that APOE4 inheritance shifts metabolism away from carbohydrates and toward lipids and ketones [9].

This metabolic shift was preserved in the hippocampus, in which APOE4 females experienced a significant rise in fatty acid oxidation transcripts required to metabolize ketones relative to APOE3. Along with these metabolic changes, female APOE4 mice also experienced the largest relative rises in inflammatory changes in interferon response and MHC-related genes [9]. These findings are consistent with the view that female-specific and APOE4-dependent increase in ketone metabolism, and the relative shortage of those ketones on a carbohydrate-based diet may cause increased inflammatory profile specifically in those APOE4 females.

We observe that the ketogenic diet rescues multiple aspects of memory and suppresses inflammation in APOE4 females. There are similar relationships among mice and humans with respect to APOE4-dependent inflammation. When mice inherit humanized APOE4 vs. APOE3, they show a higher inflammatory status in the periphery [33], and in the brain [34–36], just as is seen in humans that carry the APOE4 allele [38–40]. Hyperinflammation in brain has been proposed to contribute to Alzheimer’s disease [41], so ameliorating brain inflammation could improve memory outcomes in APOE4 humans.

Moreover, in mice, inheritance of human APOE4 causes higher levels of blood ketones to be produced in females and causes increased expression of transcripts of fatty acid oxidation in their hippocampi [9]. Similarly, in humans, APOE4 inheritance changes brain bioenergetics [4–6, 8], and induces the BHB transporter protein MCT2 and the BHB-metabolizing proteins SCOT and AACS in their brains [7]. One implication is that APOE4 inheritance in humans increases reliance on ketones in both sexes, but this reliance may be stronger in females, suggesting that females could derive greater benefit from a KD.

While we previously did not observe an increase in fat or body weight with KD in aged male mice [10] or in male or female PSAPP mice [13], here we found that KD significantly increased both. The increase we see here in fat and body weight in the APOE4 mice may represent a difference in lipid partitioning or lipid metabolism resulting from APOE4 inheritance. Others have shown that APOE4 mice weigh less and have smaller epididymal fat pads compared to APOE3 mice [45] when fed a high-fat Western diet. This inverse effect on body weight and fat between a Western (high fat and cholesterol with carbohydrates) and a ketogenic (high fat, high cholesterol, and almost no carbohydrate) diet requires additional research to determine which diet components influence body composition in APOE4 mice.

While this study provides compelling evidence of the benefits of the KD in APOE4 mice, several limitations should be noted. First, the study was conducted in a single APOE4 mouse model, and it remains unclear whether these findings will translate to humans. Second, the mechanisms of KD’s female-specific benefit are not yet fully understood and warrant further investigation. Finally, the long-term effects of the KD on brain health and cognitive function in aging populations need to be explored through clinical trials.

In conclusion, since APOE4 women experience high AD risk and a KD has been used in multiple clinical trials [46], our study suggests that a KD may provide significant benefit to synaptic plasticity, cognition, and inflammation in women bearing APOE4.

## Supplementary Information

Below is the link to the electronic supplementary material.

ESM1 (XLSX.9.59 KB)

ESM2 (XLSX.10.1 KB)

ESM3 (XLLSX.91.4 KB)

ESM4 (PDF.384 KB)

## Data Availability

RNA-seq data are deposited on the GEO repository and are accessible with the number GSE301153.
