# Supplementary figures and images for: A ketogenic diet improves memory in females in the APOE4 mouse model of Alzheimer’s disease
Source: GeroScience. 2025 Nov 24;48(2):1937–54. doi: 10.1007/s11357-025-01998-8 (PMC12972429; doi:10.1007/s11357-025-01998-8)

Supp Fig. 1

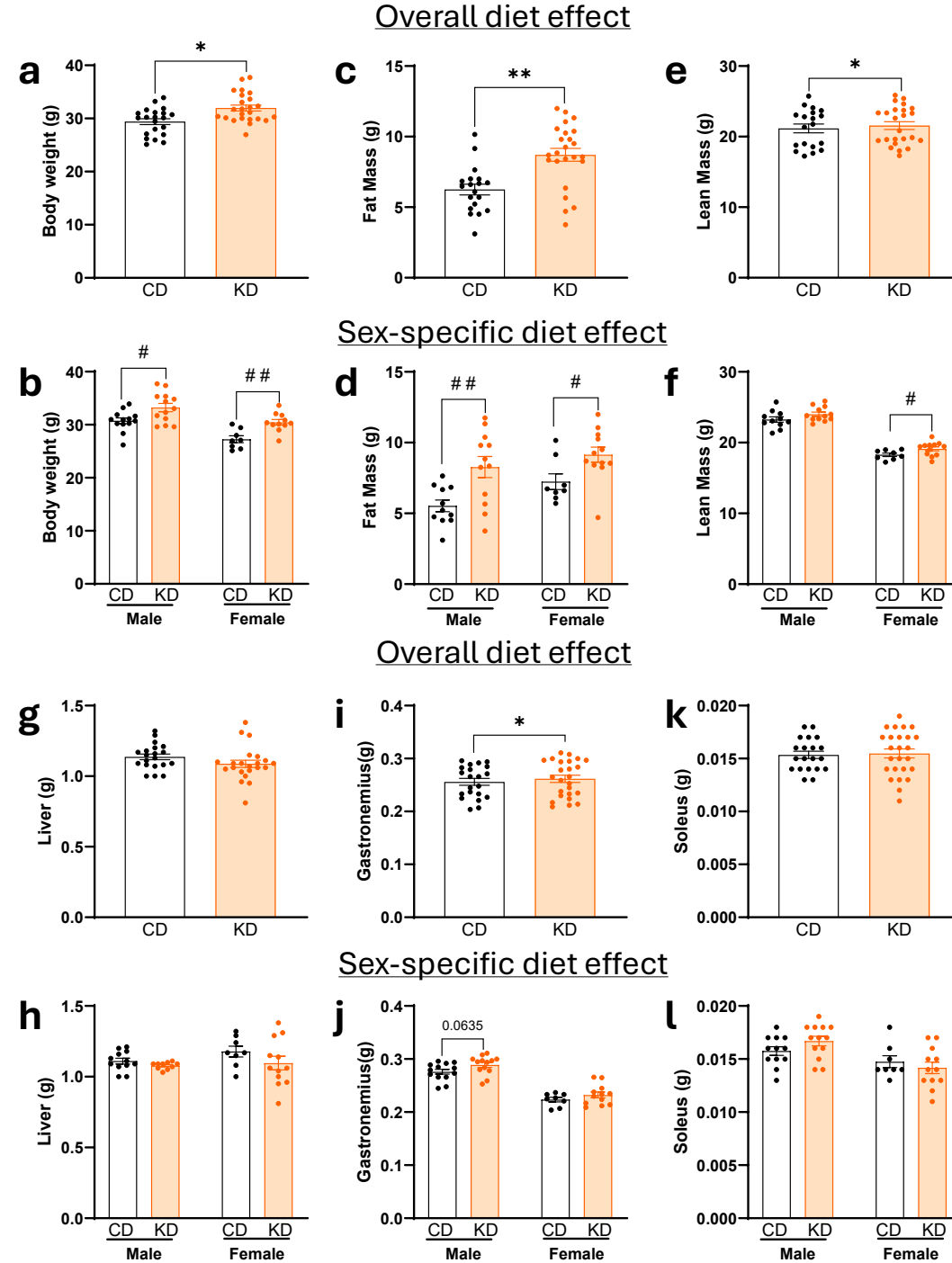

Supp Fig. 2

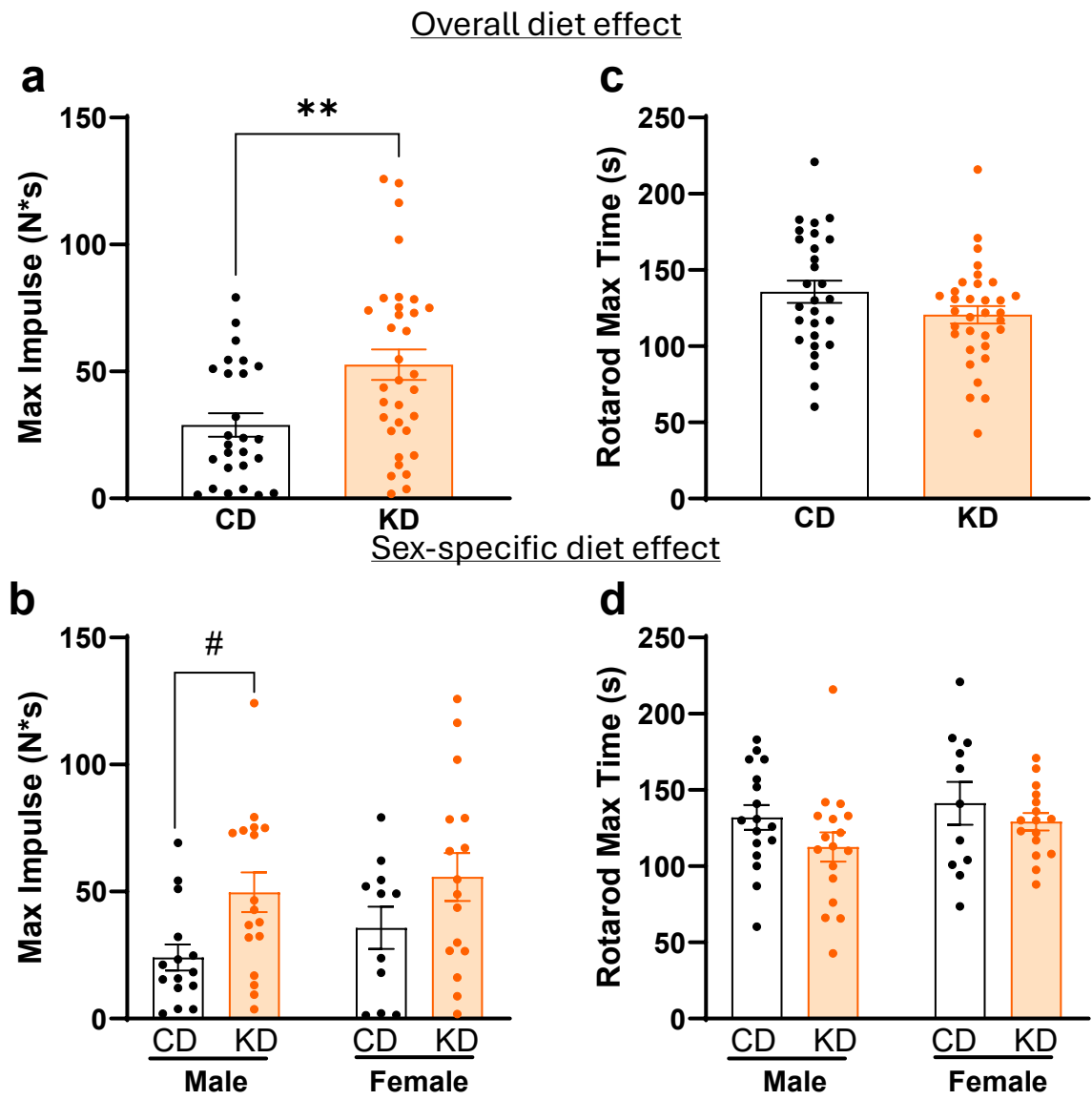

Supp Fig. 3

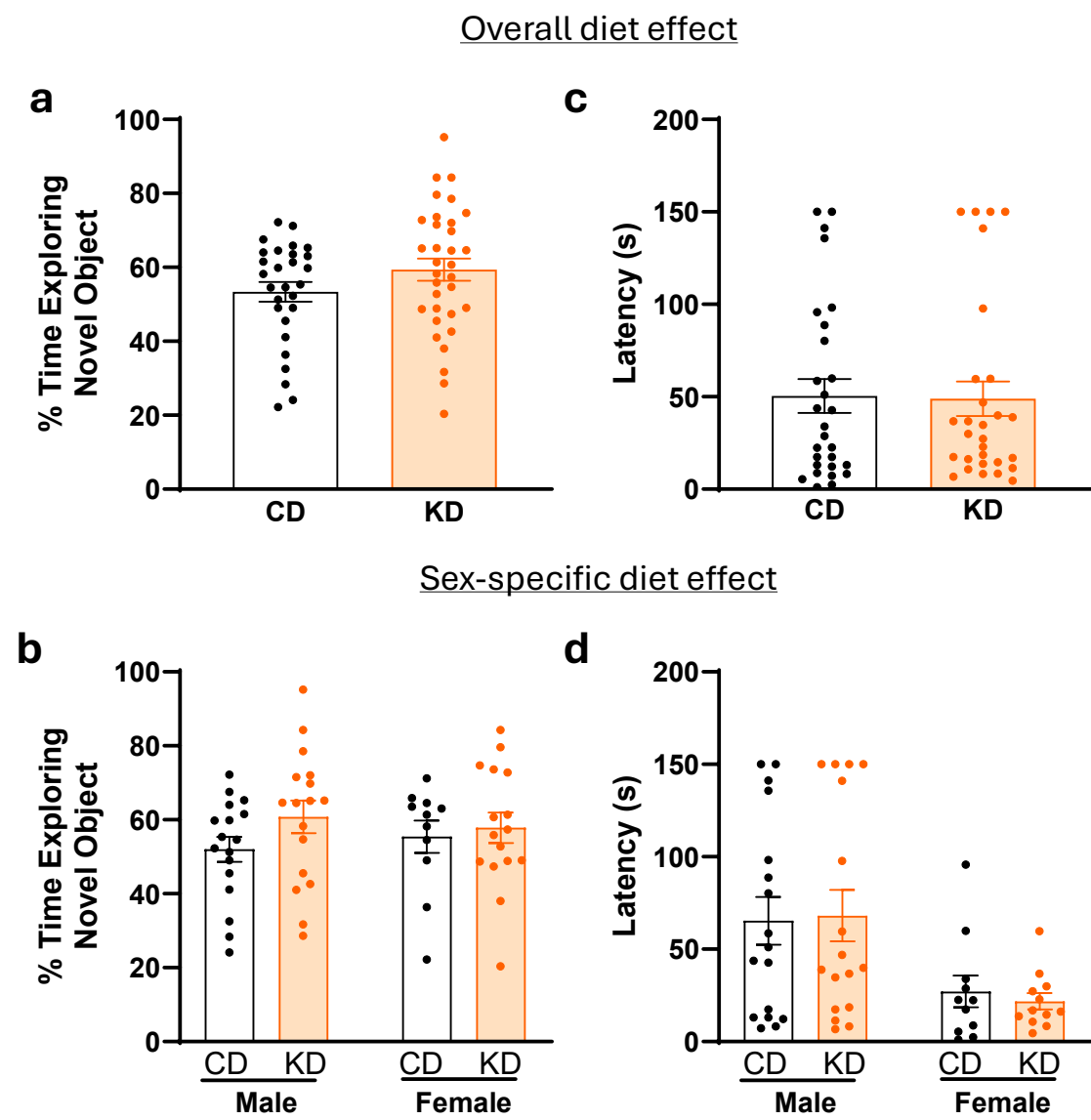

Supp Fig. 4

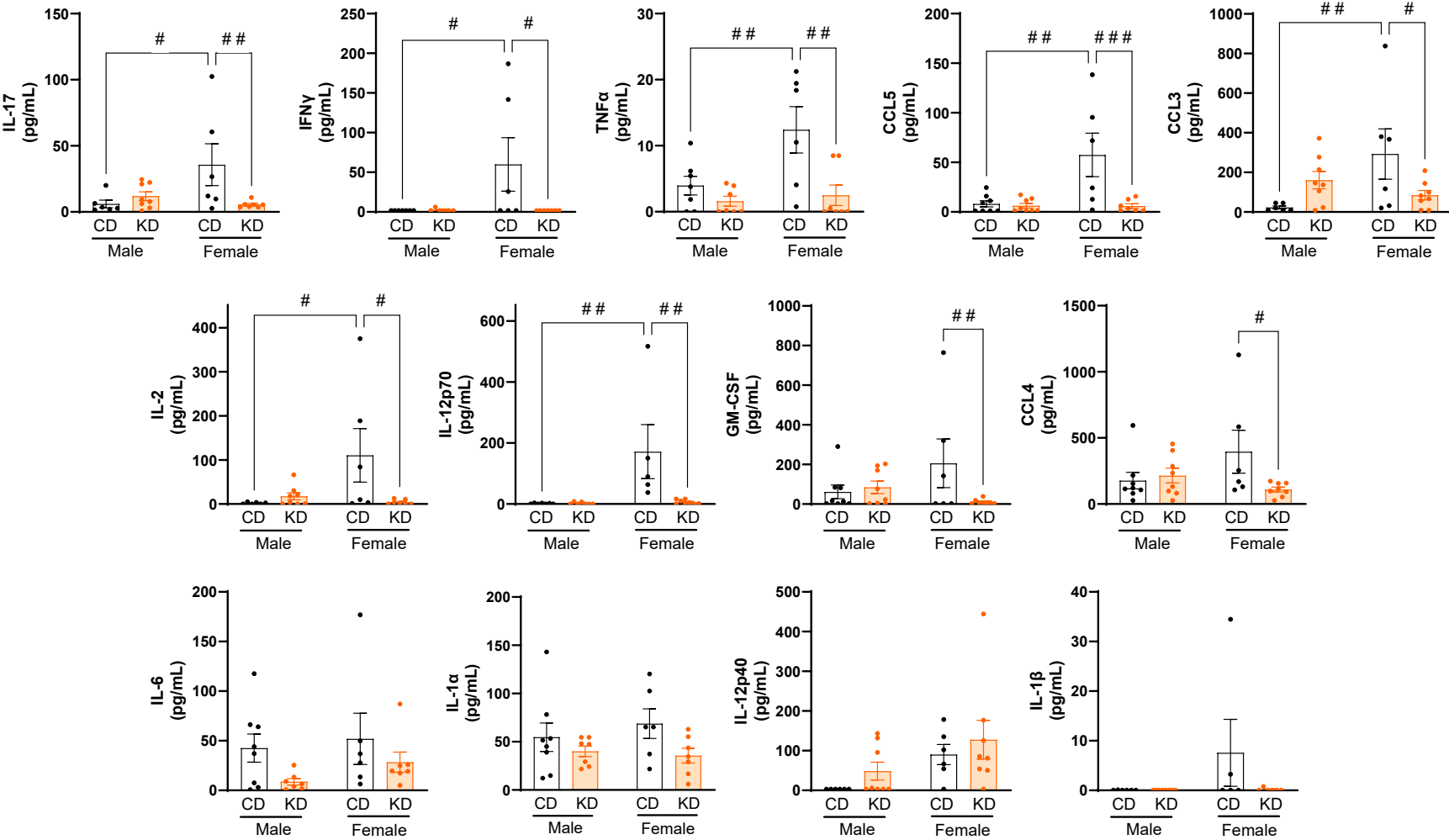

Supplement: Supplementary file 4 — (PDF.384 KB) [file 11357_2025_1998_MOESM4_ESM.pdf]
